# Supplementary material for: Establishment of an Intradermal Canine IL-31-Induced Pruritus Model to Evaluate Therapeutic Candidates in Atopic Dermatitis
Source: Vet Sci. 2023 May 4;10(5):329. doi: 10.3390/vetsci10050329 (PMC10223001; doi:10.3390/vetsci10050329)
Supplement: Supplementary file 1 [file vetsci-10-00329-s001.zip › vetsci-2324784-supplementary.pdf]

Supporting Information

Table S1. Ethogram itch behavior definitions were used to record behaviors per minute during video analysis by independently trained viewers to evaluate the canine intradermal IL-31-induced pruritic model. Example videos were also provided to visually display behavior descriptions for each viewer before the video analysis.

**Itch Behaviour Catalogue**	
**Term**	**Definition**
Head Shaking	Rapid movement of the head from one side to the other from the median line of the body. Neck muscles utilized to complete this movement. Enough force is used to complete the motion that the ears follow the motion, given the pinnae are long enough. Can be rapid and last less than one second.
Licking	Tongue contacts the skin and fur, and the head makes a motion with the neck muscles to move the tongue along any part of the dog's body. Lasts greater than one second.
Chewing/Biting	Mouth contacts any other part of the dog's body and a chewing motion is visible, utilizing the masseter muscles and visibly moving the temporal muscles of the head. Sometimes this motion involves a rocking motion of the entire body. Lasts greater than one second.
Scratching	Toes and claws from the limb (usually the hind limb) are utilized by the limb muscles to drag the claws along the skin of another part of the body multiple times. Lasts greater than one second.
Pawing	Toes and claws from the limb (usually the forelimb) are utilized by the limb muscles to drag the claws along the skin of another part of the body (usually the face). Usually done multiple times but can be one long pawing motion. Generally, lasts greater than one second, but can be faster.
Scooting	Back is hunched to allow contact of the anus to the floor. Hind limbs usually lose contact with the floor in this sitting motion. Front limbs are utilized to help drag anus along the floor in a forward motion. Tail can either be laying on the floor behind the rear or erect into the air. Lasts greater than one second.
Tail Chasing	Head is turned towards the tail and the body naturally follows. Mouth contacts some part of the body (anus, rear, tail for ex). Full 360 degree rotations can be made, but do not need to be made to qualify for this behaviour. Lasts greater than one second.
Rolling	Entire body ends up in lateral recumbency and the ventral abdomen is twisted up towards the air. The roll can than twist the body entirely to the other side from which lateral recumbency began, or may move back to the side it started. This behaviour is often, but not necessarily, associated with other itch behaviours.
Rubbing	Any part of the body is moved along the ground, either in a back and forth motion or just one direction, for greater than 2 seconds. Other muscles of the body are utilized to achieve this motion (intention). Behaviour is similar to scooting, except instead of the rectum making contact with the ground it is another part of the body (sides, abdomen, e.g.)

Table S2. Ethogram itch behavior definitions specific to intradermal injection (IDT) site of pruritogen used to record site-specific itch behaviors during video analysis by independent trained viewers

**Localized Itch Behaviours (to IDT site)**	**Definition**
**Term**	
Site Licking	Tongue contacts the skin and fur, and the head makes a motion with the neck muscles to move the tongue along the specific site of injection. Lasts greater than one second.
Site Chewing/Biting	Mouth contacts the specific site of injection and a chewing motion is visible, utilizing the masseter muscles and visibly moving the temporal muscles of the head. Sometimes this motion involves a rocking motion of the entire body. Lasts greater than one second.
Site Scratching	Toes and claws from the limb (usually the hind limb) are utilized by the limb muscles to drag the claws along the site of injection multiple times. Lasts greater than one second.
Site Rubbing	The site of injection on the body is moved along the ground, either in a back and forth motion or just one direction, for greater than 2 seconds. Other muscles of the body are utilized to achieve this motion (intention). Behaviour is like scooting, except instead of the rectum contacting the ground it is another part of the body (in this case the injection site)
Site Rolling	Entire body ends up in lateral recumbency and the ventral abdomen is twisted up towards the air. The roll can then twist the body entirely to the other side from which lateral recumbency began or may move back to the side it started. This behaviour is often, but not necessarily, associated with other itch behaviours. For this behaviour to qualify as a site roll, the injection site must contact the ground at some point during the displayed behaviour.
